# Investigation of the Effects of Salinity Exposure on Immune Defense, Morphology, and Gene Expression in the Gills of *Macrobrachium nipponense*

**DOI:** 10.3390/antiox14060655

**Published:** 2025-05-29

**Authors:** Shubo Jin, Rong Zhou, Hongtuo Fu, Wenyi Zhang, Hui Qiao, Yiwei Xiong, Sufei Jiang

**Affiliations:** 1 Wuxi Fisheries College, Nanjing Agricultural University, Wuxi 214081, China; jinsb@ffrc.cn (S.J.);; 2 Key Laboratory of Freshwater Fisheries and Germplasm Resources Utilization, Ministry of Agriculture and Rural Affairs, Freshwater Fisheries Research Center, Chinese Academy of Fishery Sciences, Wuxi 214081, China

**Keywords:** *Macrobrachium nipponense*, salinity, gill, immune response, gene response

## Abstract

*Macrobrachium nipponense* is an important economic freshwater species in China. Previous research has found that *M. nipponense* can reproduce under salinity conditions of 10 parts per thousand (ppt) and exhibits a strong ability to adapt to salinity changes in the aquatic environment. The aim of the present study was to identify the molecular mechanism of *M. nipponense* in terms of saline acclimation by identifying changes in immune response, morphology, and gene expression in the gills under a salinity of 10 ppt. The findings revealed that salinity exposure dramatically stimulated the activities of MDA, Ca^2+^Mg^2+^-ATPase, and CAT, reaching a peak on Day 7 (*p* < 0.05), indicating that these antioxidant enzymes play essential roles in protecting the body from the damage caused by saline treatment. In addition, we found no obvious morphological changes in the gills, indicating that *M. nipponense* can adapt well to water environments with such salinity. Transcriptome profiling analysis identified 168, 434, and 944 differentially expressed genes (DEGs) when comparing S0 vs. S1, S1 vs. S4, and S4 vs. S7, respectively. Furthermore, lysosome, apoptosis, amino sugar, and nucleotide sugar metabolism; the cGMP-PKG signaling pathway; pancreatic secretion; and the calcium signaling pathway represented the main enriched metabolic pathways of DEGs in the present study. Lysosome, apoptosis, amino sugar, and nucleotide sugar metabolism and the cGMP-PKG signaling pathway are immune-related metabolic pathways, while pancreatic secretion is an energy-metabolism-related metabolic pathway, suggesting that the immune response and energy metabolism play essential roles in the regulation of saline acclimation in this species. The results from the quantitative real-time PCR analyses of the DEGs were consistent with those from RNA-Seq, indicating the accuracy of the present study. This study provides valuable evidence for the acclimation of *M. nipponense* to high-salinity aquatic environments, thus indicating the potential for this species to be used in aquaculture programs in saline and alkaline water regions.

## 1. Introduction

Saline–alkaline water resources are widely distributed in inland areas, representing a global water resource with a low yield of both aquatic animals and crops. The area of saline–alkaline water resources in China is approximately 9.91 × 10^7^ hectares, ranking third globally and accounting for 10% of the world’s saline–alkaline water resources. These waters are widely distributed in the northeastern, northern, and southwestern regions of China, including coastal areas. Compared to those of seawater, saline–alkali water has the characteristics of high pH, high carbonate alkalinity, high water mineralization, diverse hydrochemical types, complex ion composition, lack of constant ion ratio, and poor water quality buffering capacity, making it unsuitable for use as drinking water and in agricultural irrigation [1,2]. Previous studies reported that high saline and alkaline levels have negative effects on the survival of organisms in water bodies. For example, chloride is the main ion that determines the salinity level in water bodies and has an inverse relationship with biodiversity in the water environment, excluding less tolerant species. A reasonable explanation for this is that increasing salinity has acute effects during specific life stages of organisms [3,4]. Thus, aquatic animals with weak saline–alkaline tolerance cannot normally reproduce in such environments, leading to low aquaculture yields in such areas. Although China has abundant saline–alkaline water resources, the utilization rate of these resources is less than 2%, indicating that they are not being effectively utilized [5,6].

Osmotic homeostasis within the cells of decapod crustaceans remains stable in the freshwater environment. Recent publications indicated that efficient cell volume regulation mechanisms (IIR: iso-osmotic intracellular regulation) play essential roles in the adaptation of decapod crustaceans to changes in external saline concentrations [7]. Under this mechanism, the extracellular fluid (ECF) is diluted when salinity decreases, which leads to an influx of water and causes the swelling of tissue cells. This is compensated by the process of regulatory volume decrease (RVD), whereby solutes (inorganic and organic) are discharged from the cells to the ECF. However, ECF osmolality increases in highly saline water environments, inducing the efflux of water from cells to the ECF and, finally, causing the cells to shrink. This is complemented by the regulatory volume increase (RVI) mechanism in which solutes (inorganic and organic) flow from the ECF into cells, restoring cell volume [8,9].

Previous publications indicated that penaeid species exhibit hyper- and hypo-osmotic regulation when the salinity is lower or higher than the iso-osmotic value, respectively [10,11]. The iso-osmotic point varies considerably in Penaeidae crustaceans, ranging from 671.3 mOsm/kg (21.1 parts per thousand, ppt) in the case of *Litopenaeus vannamei* [12] to 824 mOsm/kg (26.8 ppt) in the case of *Litopenaeus setiferus* [13]. However, Palaemonid shrimps have been found to have originally immigrated from marine to freshwaters via independent events [14,15]. The *Macrobrachium* genus includes freshwater prawns and shrimps, which exhibit hyper-osmotic regulation in freshwater and water with low salinity, and hypo-osmotic regulation in water environments with high salinity (except *M. equidens* and *M. olfersii*) [16]. Previous studies have identified the effects of salinity on the growth and development of some species, including *Macrobrachium nipponense* [17], *M. rosenbergii* [18], *Penaeus monodon* [19], and *Nephrops norvegicus* [20].

*M. nipponense* (Crustacea; Decapoda; Palaemonidae) is an important freshwater prawn species in China and is widely distributed in freshwater and low-salinity estuarine regions in the country. The annual production of *M. nipponense* reached 226,392 tons in 2023, producing huge economic benefits. The main regions for *M. nipponense* aquaculture are Jiangsu province, Anhui province, Zhejiang province, and Jiangxi province, with an annual production of more than 200 thousand tons [21]. A previous study found that the *Lc50* values of saline tolerance in juvenile *M. nipponense* with a body length of 2.0–2.5 cm and average body weight of 0.687 g were 30.71 ppt for 24 h, 26.66 ppt for 48 h, 26.31 ppt for 72 h, and 25.80 ppt for 96 h [22]. These values are similar to those of *Procambarus clarkii* (31.74 ppt for 24 h) [23], but higher than those of *M. rosenbergii* (19.33 ppt for 24 h) [24].

Gills are important organs in aquatic animals whose main function is to absorb oxygen from the water environment. The surface of the gill filaments is covered by blood vessels, and these vessels promote the absorption of oxygen from the water environment into the blood for respiratory function. In the present study, we examined the effects of salinity exposure on the gills of *M. nipponense* through histological observations, the measurement of antioxidant enzymes, and transcriptome profiling analysis after treatment under a saline concentration of 10 parts per thousand (ppt) for 0 days, 1 day, 4 days, and 7 days. This study provides valuable evidence for saline tolerance in *M. nipponense*, which could enhance the aquaculture of this species in saline and alkali regions.

## 2. Materials and Methods

### 2.1. Tissue Collection

A total of 150 healthy prawns with body weights of 3.53 ± 0.82 g were provided by the Dapu *M. nipponense* Breeding Base in Wuxi, China (120°13′44″ E, 31°28′22″ N). The prawns were maintained under laboratory conditions with a water temperature of 26.0 °C ± 1.2 and dissolved oxygen of >6.0 mg/L for 3 days prior to the saline treatment. Previous research indicated that the maximum saline concentration for *M. nipponense* in wild water resources is 10 ppt, as observed in Jingtai, Gansu province [25]. Thus, the saline concentration in the water was adjusted to 10 ppt by adding NaCl to water with a temperature of 26.0 °C ± 1.2, pH of 7.69–8.13, and a dissolved oxygen level of >6.0 mg/L. The saline concentration was measured using a salinity meter (Delixi, Wenzhou, China). The prawns were placed into this water environment, and their gills were collected at 0 days (S0), 1 day (S1), 4 days (S4), and 7 days (S7) of saline exposure. Three gills were collected and stored in 4% paraformaldehyde until histological slicing and observations were conducted. Five gills were collected and pooled together to form a biological replicate. Three biological replicates were prepared for the transcriptome profiling analysis, quantitative real-time PCR (qPCR), and examination of changes in antioxidant enzymes. The tissue samples were immediately stored at −80 °C after collection in order to prevent the degradation of RNA, and were kept under these conditions until experimental analysis.

### 2.2. Measurement of Antioxidant Enzyme Activity

A commercial kit from Nanjing Jiancheng Bioengineering Institute (Nanjing, China, the commercial kits were used as followed: SOD: A001-1, MDA: A003-1, GSH-PX: A005-1, GSH: A006-1-1, CAT: A007-1-1, T-AOC: A015-1, Na^+^K^+^-ATPase: A070-2, Ca^2+^Mg^2+^-ATPase: A070-3) was used to measure the changes in antioxidant enzymes after exposure to a saline concentration of 10 ppt. The antioxidant enzymes measured in the present study included total antioxidant capacity (T-AOC), superoxide dismutase (SOD), malondialdehyde (MAD), catalase (CAT), glutathione (GSH), glutathione peroxidase (GSH-PX), Na^+^K^+^-ATPase, and Ca^2+^Mg^2+^-ATPase. A microplate reader (Bio-Rad iMark, San Francisco, CA, USA) was used to measure all of the antioxidant indexes, following the manufacturer’s instructions.

### 2.3. Histological Observation

Hematoxylin and eosin (HE) staining was used to measure the morphological changes in the gills after exposure to the saline treatment at a concentration of 10 ppt. The three gills collected at each time point were sliced (three biological replicates), and two slices were prepared from each gill (two technique replicates). Details of the HE staining procedure have been well described in previous studies [26,27]. Briefly, the collected gills were dehydrated using different concentrations of ethanol, and then different percentages of a xylene/wax mixture were used to render the gill tissues transparent and embed the dehydrated gills. A slicer was used to slice the embedded gills into 5 µm thick sections (Leica, Wetzlar, Germany), and HE was finally used to stain the slices for 3–8 min. The slides were observed using an Olympus SZX16 microscope (Olympus Corporation, Tokyo, Japan).

### 2.4. Transcriptome Profiling Analysis

The changes in the gills’ gene expression caused by the saline treatment were identified using transcriptome profiling analysis, performed on an Illumina Hiseq-2500 sequencing platform (Illumina, San Diego, CA, USA). RNAiso Plus Reagent (TaKaRa, Osaka, Japan) was used to extract the total RNA from the gills of each biological replicate, following the manufacturer’s instructions. The total RNA concentration was measured using a spectrophotometer (Eppendorf, Hamburg, Germany), and the integrity of the extracted total RNA was measured using a 2100 Bioanalyzer (Agilent Technologies, Inc., Santa Clara, CA, USA) with an RNA integrity number (RIN) of >7.0. The procedures for the RNA-Seq and bioinformation analysis have been described in detail in previously published papers [28,29]. Briefly, a total of 4 µg of total RNA was used to construct the library, and an Illumina Hiseq-2500 sequencing platform was used to conduct the sequencing under the parameter of PE150.

Fastp software (version 0.20.0) was employed to remove low-quality raw reads with default parameters [30]. The HISAT2 software (version 0.20.0) was then employed to map the obtained clean reads to the *M. nipponense* reference genome (GenBank accession number: GCA_015104395.2) [31]. Genes were annotated in the Gene Ontology (GO) (http://www.geneontology.org/, accessed on 17 May 2024) [32], Cluster of Orthologous Groups (COG) (http://www.ncbi.nlm.nih.gov/COG/, accessed on 17 May 2024) [33], and Kyoto Encyclopedia of Genes and Genomes (KEGG) (http://www.genome.jp/kegg/, accessed on 17 May 2024) [34] databases, using an E-value of 10^−5^ [28]. Gene expression was calculated using the FPKM method, where FPKM = cDNA fragments/mapped fragments (millions)/transcript length (kb), using HTSeq-count [35]. DESeq2 was used to perform the differential expression analysis [36]. The Benjamini–Hochberg correction method was used to calculate the false discovery rate (FDR) [37] with a q-value < 0.05. A fold change > 2.0 was considered to indicate upregulated differentially expressed genes (DEGs), and a fold change < 0.5 was considered to indicate downregulated DEGs.

### 2.5. qPCR Analysis

The accuracy of the RNA-Seq was verified via qPCR. Details of the qPCR analysis procedure have been well described in previous studies [38,39]. A commercial kit from Shanghai Sangon Company (Shanghai, China) (UNlQ-10 Column TRIzol Total RNA Isolation Kit) was used to extract the total RNA from the gills obtained at each time point. The concentration of extracted total RNA was measured using a spectrophotometer (Eppendorf, Hamburg, Germany), and the integrity of the total RNA was measured using 1.2% agarose gel. The cDNA template was synthesized from 1 μg total RNA using a commercial kit (PrimeScript™ RT reagent kit, Takara Bio Inc., Kusatsu, Japan), following the manufacturer’s instructions. UltraSYBR Mixture (CWBIO, Beijing, China) was used to measure the expression level of each tissue sample according to the manufacturer’s instructions. The Bio-Rad iCycler iQ5 Real-Time PCR System (Bio-Rad, San Francisco, CA, USA) was used to conduct the qPCR analysis, which involved a SYBR Green RT-qPCR assay. All primers used for the qPCR analysis are listed in Table 1. The primers were designed based on the open reading frame of each gene, using the primer-blast tool from the National Center for Biotechnology Information (https://www.ncbi.nlm.nih.gov/tools/primer-blast/, accessed on 14 December 2024). The cDNA template was diluted to five different concentrations (1, 0.5, 0.25, 0.125, 0.0625) in order to verify the amplification efficiency of each primer. The amplification efficiency of the primers used in the present study ranged from 92.36% to 105.23%. The eukaryotic translation initiation factor 5A (*EIF*) was used as the reference gene for qPCR analysis in the present study, and has been proven to be stably expressed under various conditions in *M. nipponense* [40]. The 2^−ΔΔCT^ method was used to determine the relative expression levels [41].

### 2.6. Statistical Analysis

The statistical analysis of gene expressions and antioxidant enzyme activities was carried out using SPSS Statistics 23.0, with a one-way ANOVA used for estimation, followed by Duncan’s multiple range test [38,39]. *p* < 0.05 indicated statistical significance. Quantitative data were expressed as the mean ± SD.

## 3. Results

### 3.1. Measurement of Antioxidant Enzymes in Gills After Saline Treatment

**Figure 1:**
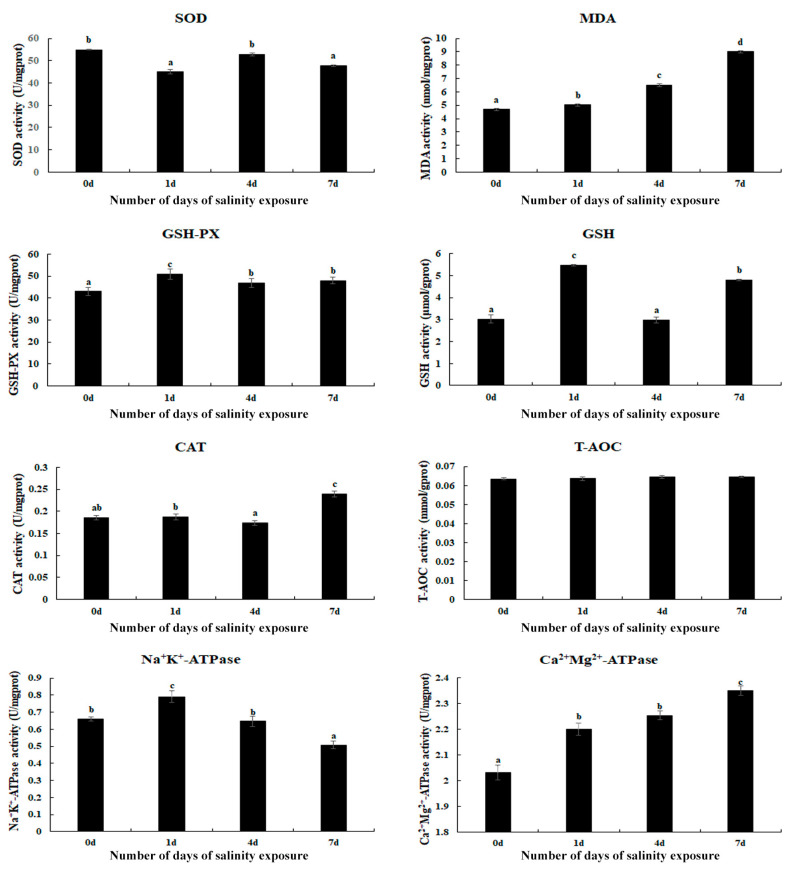
The measurement of antioxidant enzyme activities in the gills after different numbers of days of salinity exposure under a concentration of 10 ppt. Data are shown as the mean ± SD (standard deviation) of tissues from three biological replicates. Letters indicate the significant difference in the activities of antioxidant enzymes between different lengths (in days) of alkalinity exposure. CAT: catalase; GSH: glutathione; GSH-PX: glutathione peroxidase; MDA: malondialdehyde; SOD: superoxide dismutase; T-AOC: total antioxidant capacity.

Changes in the antioxidant enzymes in the gills of *M. nipponense* were measured on Day 1, Day 4, and Day 7 of saline treatment (Figure 1). The activities of MDA and Ca^2+^Mg^2+^-ATPase gradually increased with an increase in the treatment time and reached a peak on Day 7 (*p* < 0.05), while the T-AOC activities exhibited no difference between the different time points (*p* > 0.05). In addition, the saline treatment also stimulated the activities of GSH-PX, GSH, Na^+^K^+^-ATPase, and CAT. The activities of GSH-PX, GSH, and Na^+^K^+^-ATPase reached a peak on Day 1 of the saline treatment, while CAT exhibited the highest activity on Day 7 (*p* < 0.05).

### 3.2. Morphological Changes in Gills Caused by the Saline Treatment

**Figure 2:**
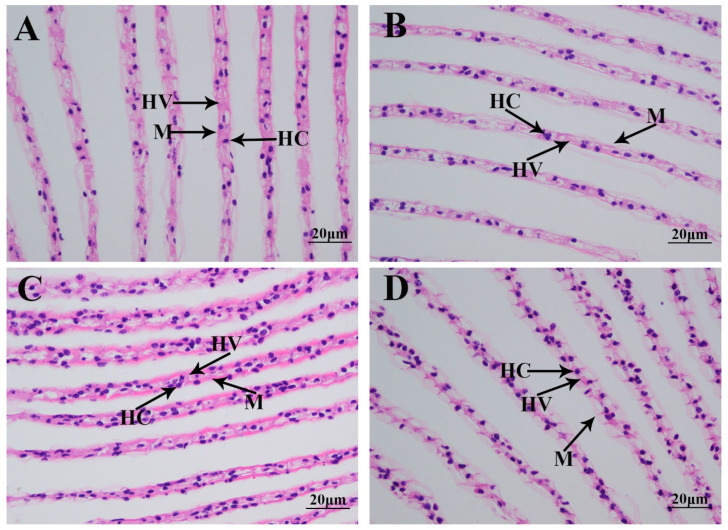
The morphological changes in the gills under a salinity of 10 ppt. HC: hemocytes; HV: hemolymph vessel; M: membrane. Scale bars = 20 µm. (**A**): the morphology of the gills without salinity exposure; (**B**): the morphology of the gills after 1 day of salinity exposure; (**C**): the morphology of the gills after 4 days of salinity exposure; (**D**): the morphology of the gills after 7 days of salinity exposure.

Morphological changes in the gills caused by the saline treatment were investigated using HE staining (Figure 2). A histological observation revealed that normal gills comprise hemocytes, hemolymph vessels, and a membrane. According to Figure 2, no obvious damage caused by the saline treatment was observed in the gills of *M. nipponense*. However, the gill tissues were found to be more swollen on Day 7 after saline treatment than those of the other time points.

### 3.3. Transcriptome Profiling Analysis

The DEGs in the gills of *M. nipponense*, caused by the saline treatment, were identified using the criterion of >2.0 for upregulated genes and <0.5 for downregulated genes. A total of 168 (103 upregulated and 65 downregulated), 434 (378 upregulated and 56 downregulated), and 944 DEGs (485 upregulated and 459 downregulated) were identified in the comparison of S0 vs. S1, S1 vs. S4, and S4 vs. S7, respectively.

**Figure 3:**
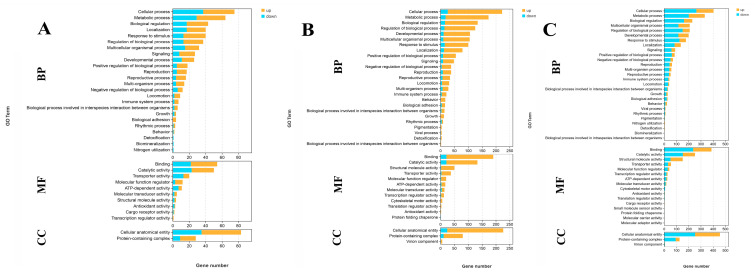
GO analysis of DEGs in the gills after different numbers of days of salinity exposure under a concentration of 10 ppt. (**A**): GO analysis of S0 vs. S1. (**B**): GO analysis of S1 vs. S4. (**C**): GO analysis of S4 vs. S7. BP: biological process; MF: molecular function; CC: cellular component.

A total of 103, 323, and 657 DEGs were identified for annotation in the GO database when comparing S0 vs. S1, S1 vs. S4, and S4 vs. S7, respectively. Cellular processes, metabolic processes, biological regulation, binding, catalytic activity, and cellular anatomical entity represent the main enriched functional groups in the GO analysis of these three comparisons (Figure 3).

**Figure 4:**
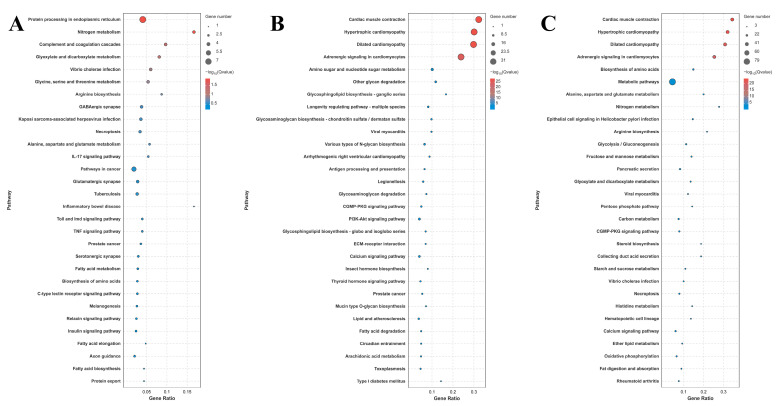
KEGG analysis of DEGs in the gills after different numbers of days of salinity exposure under a concentration of 10 ppt. (**A**): KEGG analysis of S0 vs. S1. (**B**): KEGG analysis of S1 vs. S4. (**C**): KEGG analysis of S4 vs. S7.

A total of 42, 116, and 185 DEGs were identified for annotation in the KEGG database for the comparison of S0 vs. S1, S1 vs. S4, and S4 vs. S7, respectively. Lysosome, apoptosis, amino sugar, and nucleotide sugar metabolism; the cGMP-PKG signaling pathway; the calcium signaling pathway; and pancreatic secretion represent the main enriched metabolic pathways in the KEGG analysis of these three comparisons (Figure 4).

### 3.4. Identification of Candidate Genes Involved in the Regulation of Saline Acclimation

A total of 15 DEGs were selected from the main enriched metabolic pathways, which were differentially expressed in at least two comparisons (Table 2). Thus, these DEGs were considered candidate genes involved in the regulation of saline acclimation in *M. nipponense*. Among these DEGs, only four were differentially expressed between S0 and S1, and their expressions were downregulated. Moreover, 13 DEGs were differentially expressed between S1 and S4, of which 12 were upregulated, and 14 DEGs were differentially expressed between S4 and S7, of which 11 were downregulated. Notably, the expressions of legumain and chitinase continuously increased after salinity exposure, while cathepsin B showed a continuously decreasing trend.

### 3.5. qPCR Analysis

**Figure 5:**
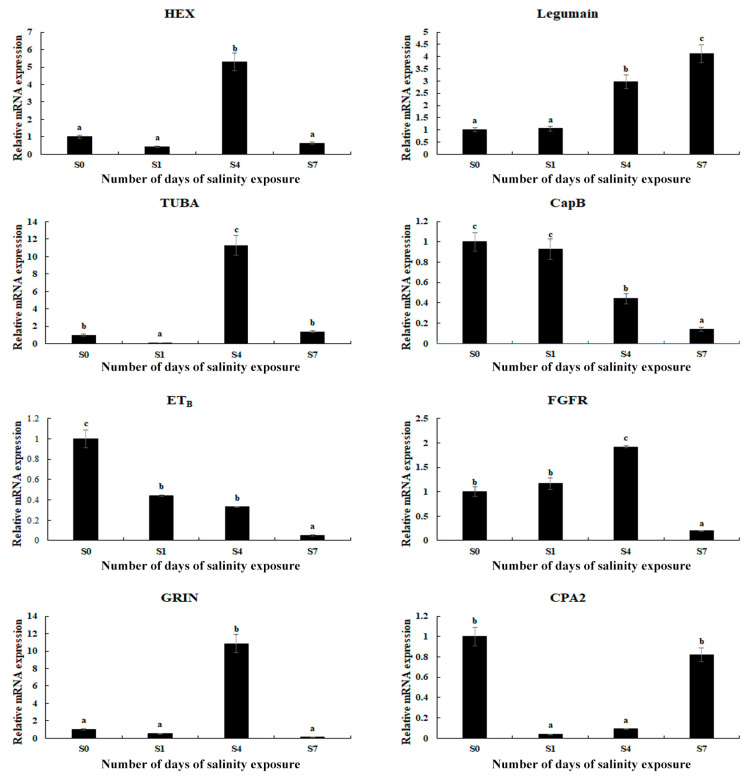
Verification of the DEGs’ expression in the gills by qPCR analysis after different numbers of days under a saline treatment of 10 ppt. Data are shown as the mean ± SD (standard deviation) of tissues from three biological replicates. Letters indicate significant differences between different numbers of days.

The expressions of eight selected DEGs were measured using qPCR in order to verify the accuracy of the RNA-Seq (Figure 5). The relative expressions of the DEGs at S0 were set as 1, and the expressions at the other time points were compared with S0. The results of the qPCR analysis of the DEGs were consistent with those of the RNA-Seq, indicating the accuracy of the present study. The expressions of hexosaminidase (*HEX*), α-tubulin (*TUBA*), fibroblast growth factor receptor 1 (*FGFR*), and glutamate receptor ionotropic (*GRIN*) reached a peak on Day 4 of the saline treatment (*p* < 0.05). In addition, saline treatment stimulated the expression of legumain, while the expression of cathepsin B (*CapB*) and endothelin receptor type B (*ET_B_*) decreased with the increase in salinity.

## 4. Discussion

*M. nipponense* exhibits a dramatically strong ability to adapt to changes in saline concentrations in the water environment [25]. Gills are important organs in aquatic animals, absorbing oxygen from water and playing an essential role in the resistance to stress in aquatic environments [42]. The aim of the present study was to examine the regulatory molecular mechanism of *M. nipponense* in terms of its ability to adapt to changes in saline concentrations by investigating changes in antioxidant enzymes, morphology, and gene expression in the gills under a saline concentration of 10 ppt.

Environmental changes can stimulate the production of reactive oxygen species (ROS), which can destroy the normal structure of cells, leading to morphological changes in the tissues of aquatic animals [43]. Previous studies found that ROS can be continuously eliminated from aquatic animals by the antioxidant enzyme defense system, protecting animals from damage caused by environmental changes [44,45]. The main antioxidant enzymes include MDA, CAT, SOD, and GSH. Previous studies indicated that the antioxidant enzyme defense system exhibits dramatic differences in response to the stress of saline treatments depending on the species, salinity tolerance, and duration of exposure. For example, the activities of SOD and glutathione reductase were reported to be stimulated in the gills of *Scylla serrata* when the salinity increased from 15 ppt to 35 ppt, while decreased activities were identified in CAT and GSH-PX [43]. The activities of SOD, CAT, GSH-PX, and Na^+^K^+^-ATPase were found to change when *Litopenaeus vannamei* were subjected to an acute salinity change [46]. GSH-PX and glutathione-S-transferase were reported to play essential roles in the detoxification of ROS, responding to salinity changes in *Paralichthys olivaceus* [47]. In the present study, saline treatment significantly stimulated the activities of MDA, CAT, and Ca^2+^Mg^2+^-ATPase in the gills of *M. nipponense*, reaching a peak on Day 7 under a saline concentration of 10 ppt, suggesting that these antioxidant enzymes play essential roles in protecting gills from damage caused by saline exposure in this species. MDA is an essential product of lipid peroxidation, mainly produced from polyunsaturated fatty acids in the cell membrane under the attack of ROS and free radicals [48]. Lipid peroxidation can damage the normal structure and function of cell membranes and can lead to a series of physiological disorders, including cell apoptosis and inflammatory reactions [49,50]. Catalases exhibit catalytic activity through the dismutation of H_2_O_2_ into water and molecular oxygen [51]. In addition, catalases also have many additional functions, including the decomposition of peroxynitrite [52], the oxidization of nitric oxide into nitrogen dioxide [53], and the metabolization of reactive sulfide species [54], and exhibit marginal peroxidase [55] and low oxidase activity [56]. Ca^2+^Mg^2+^-ATPase is an enzyme widely distributed throughout the biological membranes of aquatic animals. It can catalyze the hydrolysis of ATP into ADP and inorganic phosphorus, promoting the release of energy [57,58].

Previous publications have reported that aquatic animals can adapt to changes in salinity in the water environment by modulating the epithelial cells in the gills [59]. The number of chloride-secreting cells on the gill filaments and gill lamellae of *Acipenser sinensis* were observed to be significantly increased in order to survive in water with a saline concentration of 25 ppt [60]. A densely distributed microtubule system was observed in the cytoplasm of *Sebastes schlegelii*, and the number of mitochondria significantly increased in order to adapt to a highly saline water environment [61]. In another study, a highly saline and alkaline water environment resulted in the rupture of gill epithelial cells and the hyperplasia of chloride cells in *Salmoclarki henshawi* [62]. In the present study, no obvious damage was observed to be caused by exposure to saline at a concentration of 10 ppt in the gills, indicating that *M. nipponense* can survive well in water environments with such salinity. However, the gill tissues were found to be swollen on Day 7 of the saline treatment, suggesting that this change may be associated with the regulation of saline acclimation in this species.

Transcriptome profiling analyses have been performed in many aquatic animals following salinity exposure in order to select the key metabolic pathways and genes involved in saline acclimation [63,64,65,66]. These publications found that the main enriched metabolic pathways of DEGs are mainly involved in the regulation of osmotic pressure, oxidative stress, energy metabolism, and the immune response. In one study, transcriptome analysis was employed to reveal the molecular response to salinity acclimation in the gills and hepatopancreas of *M. nipponense* in water with different saline concentrations [67]. The enriched metabolic pathways of DEGs were found to be involved in the regulation of osmoregulation, energy metabolism, and the immune response [67]. In the present study, lysosome, apoptosis, amino sugar, and nucleotide sugar metabolism; the cGMP-PKG signaling pathway; the calcium signaling pathway; and pancreatic secretion represent the main enriched metabolic pathways in the gills of *M. nipponense* after exposure to a salinity concentration of 10 ppt, suggesting that these metabolic pathways and the DEGs from these pathways play essential roles in the regulation of saline acclimation in *M. nipponense*.

Lysosomes are membrane-bound organelles in eukaryotic cells that play a crucial role in cellular digestion and waste disposal [68]. Lysosomes contain many hydrolytic enzymes and their primary function is the digestion of substances that enter the cell from the outside or the digestion of the local cytoplasm or organelles in the cell. When cells become old, lysosomes rupture, releasing hydrolytic enzymes that digest the entire cell [69,70]. *HEX* and legumain are important DEGs enriched in the metabolic pathways of lysosomes. *HEX* is an important lysosomal enzyme, playing essential roles in the degradation of glycosphingolipids, glycoproteins, and other glycoconjugates in order to ensure proper cellular waste disposal and maintain lysosomal integrity [71]. Its dysregulation is closely associated with various human diseases, including neurodegenerative disorders [72] and reproductive health [73]. Proteases play essential roles in the regulation of protein turnover, the acclimation of cellular stress, and the contribution of energy metabolism [74,75]. Legumain is an important mammalian protease that cleaves substrates and participates in the regulation of many physiological and pathological processes, including kidney function, bone remodeling, cancer, and cardiovascular and neurodegenerative diseases [76].

Apoptosis refers to the orderly process of cell death, controlled by the activation and expression of a series of genes, in order to maintain the stability of the internal environment. It is an active process initiated by the cell itself in an attempt to better adapt to the surrounding environment [77]. *CapB* and *TUBA* are important DEGs enriched in the metabolic pathway of apoptosis. α-tubulin and β-tubulin combine to form heterodimers, which are required in the formation of microtubules. Microtubules are a crucial component of the cytoskeleton and are involved in a variety of cellular functions [78,79]. The detyrosination of *TUBA* is considered a vital regulatory signal for mitosis and muscle mechanotransduction [80]. The dysregulation of *TUBA* detyrosination results in various pathological disorders, including increased tumor aggressiveness [81], the onset of neuronal disorders [82], heart failure [83], and cardiomyopathy [84].

The products of amino sugar and nucleotide sugar metabolism have many physiological functions in living organisms. These products play essential roles in cells, especially in maintaining and repairing the cell wall. Amino sugars are important substances for energy metabolism and are produced through glucose metabolism. Amino sugars are involved in the regulation of signal transduction and in cell recognition within cells [85,86]. Nucleotide sugars, which are widely found in cells, are important metabolic products that play essential roles in the regulation of signal transduction, cell division, and cell apoptosis [87,88]. Chitin is a prevalent insoluble polysaccharide mainly found in the scales of fish, the exoskeleton of arthropods (insects and crustaceans), and fungal cell walls [89]. Chitinases are versatile enzymes in the degradation of chitin, making them valuable materials in both natural ecosystems and various industrial applications [90,91]. In addition, chitinases also play essential roles in the regulation of the immune response [92], molting in crustaceans [93,94], development [95], and environmental processes [91,96].

The cGMP-PKG signaling pathway is an important intracellular signaling cascade in organisms that regulates the physiological processes of smooth muscle relaxation, cardiovascular function, neuronal signaling, and cell growth [97,98]. It is primarily activated by the second messenger cyclic GMP and is involved in the activation of protein kinase G. Disorders of this pathway contribute to various diseases, including cardiovascular diseases [99], cancer [100], and pulmonary hypertension [101].

The calcium signaling pathway causes cells to transmit and regulate signals by changing the concentration of calcium ions. Calcium ions are generally considered a second messenger, forming an electrochemical gradient inside and outside the cell because of the concentration differences [102]. When the cell is stimulated, calcium ions enter it through the cell membrane, triggering a series of biochemical reactions that ultimately affect the physiological functions and gene expression of the cell [103]. *ET_B_* and Solute carrier family 8 (*SLC8A*) are enriched in both the cGMP-PKG and calcium signaling pathways, indicating that they participate in the regulation of saline acclimation in *M. nipponense*. Endothelin receptors are a class of G-protein-coupled receptors that are widely expressed in endothelial and vascular smooth muscle cells [104]. Endothelin receptors play essential roles in the regulation of vascular, renal, pulmonary, coronary, and cerebral circulation. There are two subtypes of endothelin receptors: endothelin receptor type A (*ET_A_*) and B (*ET_B_*) [105,106]. A previous study reported that both *ET_A_* and *ET_B_* mediate the activation of phospholipase (PL) A2, PLC, and PLD through diverse G protein couplings [107]. The solute carrier gene family identifies 55 distinct gene families, with at least 400 putatively functional protein-coding genes. Solute carrier family transporters (*SLCs*) are vital for the uptake of nutrients and trace elements in the placenta, playing an essential regulatory role in the process of growth and development.

The pancreas is a vital endocrine and exocrine organ, mainly functioning in the release of hormones and the secretion of fluid, electrolytes, and various enzymes involved in the digestion of food [108]. The major hormones secreted by the pancreas include insulin, glucagon, and pancreatic polypeptide [109]. Insulin and glucagon play essential roles in the process of carbohydrate and lipid metabolism, which are necessary for maintaining normal blood concentrations of glucose [110]. Cholecystokinin A receptor (*CCKAR*) and ryanodine receptor 2 (*RyR2*) are enriched both in the metabolic pathways of the calcium signaling pathway and in pancreatic secretion. *CCKAR* plays essential roles in the mediation of some gastrointestinal functions, including gallbladder contractions [111], delays in gastric emptying [112], and the control of colonic motility [113]. *RyR2* is a Ca-release channel on the sarcoplasmic reticulum (SR) membrane, where the main intracellular Ca is stored [114]. *RyR2* can be activated by a small amount of Ca when it enters the cytosol through L-type Ca channels, and *RyR2* promotes the release of a large amount of Ca from the SR into the cytosol [115]. The above evidence indicates that the physiological processes of immune response and energy metabolism play essential roles in the regulation of saline acclimation in *M. nipponense*, protecting them from the damage caused by salinity exposure.

qPCR analyses were conducted to verify the expression of the selected DEGs, and the findings were consistent with those of the RNA-Seq, indicating the accuracy of the present study. The expressions of the majority of the selected DEGs reached a peak on Day 4 after salinity exposure, and then decreased to normal levels. However, salinity exposure continuously stimulated the expressions of legumain and chitinase, indicating that they positively regulate saline acclimation in *M. nipponense*; however, this requires further investigation.

## 5. Conclusions

In conclusion, the results of the present study demonstrated that the activities of MDA, Ca^2+^Mg^2+^-ATPase, and CAT in the gills of *M. nipponense* reached a peak on Day 7 of the saline treatment, indicating that these antioxidant enzymes play an essential role in protecting from the damage caused by high salinity exposure. In addition, no obvious morphological changes were observed on the gills following exposure to a salinity of 10 ppt. The transcriptome profiling analysis revealed that lysosome, apoptosis, amino sugar, and nucleotide sugar metabolism; the cGMP-PKG signaling pathway; pancreatic secretion; and the calcium signaling pathway represented the main enriched metabolic pathways in the present study, suggesting that the immune response and energy metabolism processes play essential roles in the regulation of saline acclimation in *M. nipponense* in highly saline environments. The qPCR analyses of the DEGs verified the accuracy of the RNA-Seq in the present study. This research provides evidence for the ability of *M. nipponense* to acclimate to highly saline water environments, thus indicating the potential for this species to be used in aquaculture programs in saline and alkaline water regions.

## Figures and Tables

**Table 1:** The primers used in the present study.

Gene	Primer	Efficiency (%)
*HEX*	F: CCTTCTTCGGTGCTCGTCAT	102.34
	R: GGCGCATCAGGTCTTCCTTA	
Legumain	F: TCACTGAACCCAAACCCAGG	96.13
	R: CCCAATTCCTTCCATGGCCT	
*TUBA*	F: ACCTTCTTCAGCGAATCGGG	97.88
	R: TTGTTAGCCGCGTCCTCTTT	
*CapB*	F: ATTCCCGAATGCGAGCATCA	92.36
	R: CCTCAACGGGGCCATTAGTC	
*EDNRB*	F: ATCACCCACATGGCGTTCTT	104.64
	R: CCTCGTTCGGTGGCTCTTTA	
*FGFR*	F: CAGGCTTCAGGTTCTGAGGG	98.59
	R: CCAACTGGAGCGTCACTCTT	
*GRIN*	F: AGACGCCATCCAAGTGACAG	105.23
	R: ATTCGGTCTCCGCTCGAATC	
*CPA2*	F: GTATCAAGTCCTACGCCGGG	98.83
	R: TGAACACCTGACGTACCTGC	
*EIF*	F: CATGGATGTACCTGTGGTGAAAC	101.39
	R: CATGGATGTACCTGTGGTGAAAC	

**Table 2:** The candidate genes involved in the regulation of salinity acclimation.

Gene	Accession Number	Metabolic Pathway	Log (Flod Change)		
			S0 vs. S1	S1 vs. S4	S4 vs. S7
Sphingomyelin phosphodiesterase (*SMPD*)	ncbi_135213205	Lysosome	−3.2		5.3
Hexosaminidase (*HEX*)	ncbi_135212715	Lysosome, amino sugar, and nucleotide sugar metabolism		3.6	−3.8
Legumain	ncbi_135217338	Lysosome		1.4	1.7
Tubulin alpha (*TUBA*)	ncbi_135200421	Apoptosis		9.9	−9.9
Cathepsin B (*CapB*)	MSTRG.22967	Apoptosis		−1.2	−1.5
Chitinase	ncbi_135197868	Amino sugar and nucleotide sugar metabolism		8.7	3.2
Endothelin receptor type B (*ET_B_*)	ncbi_135219635	cGMP-PKG signaling pathway, calcium signaling pathway	−2.1		−3.3
Solute carrier family 8 (*SLC8A*)	ncbi_135203101	cGMP-PKG signaling pathway, calcium signaling pathway		8.6	−11.8
Myosin heavy chain 6/7 (*MYH*)	ncbi_135196459	cGMP-PKG signaling pathway		12.2	−11.3
Cholecystokinin A receptor (*CCKAR*)	ncbi_135216887	Calcium signaling pathway, pancreatic secretion		3.3	−3.5
Fibroblast growth factor receptor 1 (*FGFR*)	ncbi_135226152	Calcium signaling pathway		1.8	−3.0
Ryanodine receptor 2 (*RyR2*)	ncbi_135205294	Calcium signaling pathway, pancreatic secretion		5.4	−7.5
Glutamate receptor ionotropic (*GRIN*)	ncbi_135225876	Calcium signaling pathway		9.0	−9.0
Carboxypeptidase A2 (*CPA2*)	ncbi_135221766	Pancreatic secretion	−4.5		4.2
Solute carrier family 12 (*SLC12A2*)	ncbi_135195635	Pancreatic secretion	−2.3	1.2	

## Data Availability

The raw data of the present study have been submitted to NCBI with the accession numbers SRX28127557-SRX28127568. All other data are contained within the main manuscript.
